# The prevalence of malocclusion and its association with dental caries among 12-18-year-old disabled adolescents

**DOI:** 10.1186/1472-6831-14-123

**Published:** 2014-10-01

**Authors:** Sajith Vellappally, Seby J Gardens, Abdul-Aziz Abdullah Al Kheraif, Madhusudan Krishna, Suresh Babu, Mohamed Hashem, Vimal Jacob, Sukumaran Anil

**Affiliations:** Dental Health Department, Dental Biomaterials Research Chair, College of Applied Medical Sciences, King Saud University, Post Box: 60169, Riyadh, 11433 Saudi Arabia; Department of Public Health Dentistry, Mahe Institute of Dental Sciences, Union Territory of Pondicherry, Pondicherry, India; Dental Biomaterials Research Chair, College of Applied Medical Sciences, King Saud University, Riyadh, 560013 Saudi Arabia; Krisha Devaraya College of Dental Sciences, Bangalore, 562157 Karnataka India; Department of Public health Dentistry, Navodaya Dental College and Hospital, Raichur, Karnataka India; Dental Health Department, Dental Biomaterials Research Chair, College of Applied Medical Sciences, King Saud University, Riyadh, 11433 Saudi Arabia; Dental Caries Research Chair, College of Dentistry, King Saud University, Riyadh, Saudi Arabia; Department of Periodontics and Community Dentistry, College of Dentistry, King Saud University, Post Box: 60169, Riyadh, 11545 India

**Keywords:** Dental caries, Malocclusion, Disabled children, Epidemiology, Mental retardation

## Abstract

**Background:**

To assess the prevalence of malocclusion among 12-18-year-old disabled adolescents in Chennai, Tamil Nadu, India, by using the Dental Aesthetic Index (DAI) and to determine the association of malocclusion with dental caries.

**Methods:**

This cross-sectional study included 243 children with various mental disabilities with or without physical infirmities. The Dental Aesthetic Index (DAI) and the dentition status were recorded using the World Health Organization Oral Health Surveys – Basic Methods (1997) Pro-forma. The Decayed (D), Missing (M) and Filled (F) components of the DMF index were calculated using the Dentition Status and Treatment Need (DSTN). A Chi-square test, ANOVA, and t-test were used to derive inferential statistics.

**Results:**

The mean DAI score ± standard deviation was 39.0 ± 12.3. A total of 123 (50.6%) participants (74 males and 49 females) had DAI scores of 36 and above, which indicated a handicapping malocclusion requiring mandatory orthodontic treatment. Sixty-nine (28.4%) adolescents (36 males and 33 females) had DAI scores between 31 and 35, which indicated severe malocclusion, for which orthodontic intervention was desirable. Incisal segment crowding (84.8%) was the most common aspect of the malocclusion. The mean DMFT score was 4.36 ± 3.81, and 82.8% of the participants had a DMFT score > 0. There was no statistically significant correlation between the mean DAI and DMFT scores (r = 0.090, p = 0.15). Only 16 (6.6%) of the adolescents had minor or no anomaly not needing orthodontic treatment.

**Conclusions:**

The prevalence of malocclusion and dental caries was found to be high. However, there was no positive correlation between the severity of malocclusion and dental caries among the surveyed disabled adolescents.

## Background

Children with disabilities may be physically, mentally, or socially challenged and have more marked oral health problems, either because of their actual disability or because of associated medical conditions [1, 2]. According to the American Academy of Pediatric Dentistry (AAPD) children and adolescents with special health care needs, include any physical, developmental, mental, sensory, behavioral, cognitive, or emotional impairment or limiting condition that requires medical management, health care intervention, and/or use of specialized services or programs. The condition may be congenital, developmental, or acquired through disease, trauma, or environmental cause and may impose limitations in performing daily self-maintenance activities or substantial limitations in a major life activity [3].

The oral conditions of children with disabilities are reported to be worse, either due to the existing disability or due to medical, economic or social reasons. Studies have revealed that children with a disability have worse oral health and greater treatment needs than healthy children [4–8]. Caries and the premature loss of deciduous teeth may lead to malocclusion in the permanent dentition [9]. The prevalence of malocclusion is reported to be higher among physically and/or mentally disabled children compared to healthy children [10]. Although studies have investigated the prevalence of malocclusion among disabled individuals [11–13] and the association of malocclusion and caries among healthy children [14, 15], the association of malocclusion and caries among children with special health care needs (CSHCN) has not been reported to date.

In India, one-third of the total disabled population are children, and 6 -10% of children are born disabled. The oral health care needs are almost twice as high among children and adolescents with special health care needs [16]. As per the 2001 population census, approximately 2.6% of individuals are disabled in the state of Tamil Nadu in South India [17]. The data on dental diseases among disabled children are limited. The aim of the present study was to determine the prevalence of malocclusion among 12-18-year-old children with special health care needs (CSHCN) in Chennai, Tamil Nadu, India and to determine the association of malocclusion with dental caries.

## Methods

### Study population

This study was approved by the Institutional Review Board of the Ragas Dental College, Chennai, India. Permission to conduct the study was solicited and obtained from the Special Commissioner for the disabled and the Heads of schools for CSHCN in Chennai, India. The study was conducted during January to March 2001. The list of the registered special schools for disabled individuals was obtained from the office of the Special Commissioner; of the 22 institutions contacted, 8 were excluded from the study for the following reasons: One was a vocational training center for adults, 3 institutions were no longer functioning, and 4 institutions declined permission to conduct the study. Fourteen institutions accepted the study proposal and granted permission to conduct the study. Written consent was also sought from the parents/guardians prior to the oral examination of the children. All children of ages ≥ 12 and < 19 years, with an Intelligence Quotient (IQ) ≤ 85, who were present on the scheduled examination days were included in the study. Children who were not able to cooperate due to their medical conditions and those children whose parents refused to give consent were excluded from the study. Participants who had undergone any kind of orthodontic treatment earlier were also excluded from the study.

### Pilot study and examiner calibration

A pilot study involving 24 mentally challenged children at the National Institute for Empowerment of Person with Multiple Disabilities (NIEPMD), Muttukadu, Tamil Nadu, India, was undertaken to determine the feasibility of the study, the time required for reviewing medical records, for conducting oral examinations, and the applicability of World Health Organization (WHO) Oral Health Surveys – Basic Methods (1997) Pro-forma [18]. Intra-examiner calibration was performed by reexamining the 24 children included in the pilot study after 3 weeks because a single examiner (SJG) was assigned to carry out the pilot oral examinations. The intra-examiner reliability was assessed using kappa test. A kappa value of 0.82 indicating good reproducibility was obtained. The reproducibility was 90% for the Dental Aesthetic Index (DAI) scores and 87% for Decayed, Missing, Filled Teeth (DMFT) scores, respectively. A well trained recording clerk assisted the examiner throughout the oral examination procedure.

### Procedure

The demographic details of the participating children and data on their disability status, IQ, systemic diseases and history of regular medications (if any) were collected from the medical records. Type III clinical examinations, as recommended by American Dental Association (ADA) specifications [19], were performed. The standard DAI components, as described by WHO [18], were recorded using community periodontal index probe, rubber stopper and mouth mirror. The number of decayed, missing and filled teeth was also recorded using Dentition Status and Treatment Need (DSTN), and this number was converted into a DMF score. Custom-made acrylic finger caps were used as mouth props to avoid soft tissue injury to the participants and finger injury to the examiner.

### Statistical analysis

Data analysis was performed using the Statistical Package for the Social Sciences software version 16.0 for Windows (SPSS Inc., Chicago, IL, USA). The correlation between the DAI and DMFT scores was measured by Spearman rank-order correlation coefficient (r). A chi-square test, ANOVA and t-test were used to derive the inferential statistics, and a p-value of 0.05 was considered the threshold for statistical significance.

## Results

A total of 243 adolescents with mental disabilities, with or without physical infirmities, composed the study population. There were 146 (60.1%) male and 97 (39.9%) female study participants. A total of 108 children had a mental disability alone (MD), 55 had MD and cerebral palsy (MD + CP), 36 had Down’s syndrome (DS), 18 had MD and a learning disability (MD + LD), 14 had MD and autism (MD + A) and 12 had MD and a speech-hearing impairment (MD + S-HI). The mean age of the children was 14.1 ± 2.0. More than half (n = 145; 59.7%) of the adolescents lived with their parents; the remaining 40.3% (n = 98) were residents in the institutions.

The distribution of DAI scores according to gender, various disability conditions and IQ are given in Table 1. The mean DAI score for the total sample was 39.0 ± 12.3. Approximately 93% of the adolescents had DAI scores ≥ 26, thus requiring some form of orthodontic treatment or the other. A total of 123 (50.6%) participants (74 males and 49 females) had DAI scores 36 and above, which indicates a handicapping malocclusion requiring mandatory orthodontic treatment. Sixty-nine (28.4%) participants (36 males and 33 females) had DAI scores between 31 and 35, which indicated a severe malocclusion, for which orthodontic intervention was desirable. However, there were no significant differences in DAI scores between gender, among the various disability conditions or at the various levels of IQ.

**Table 1:** **Number and percentage of adolescents in DAI level according to variables (gender, disability conditions and intelligence quotient)**

Variables		Total n (%)	DAI scores n (%)			
			≤25	26–30	31–35	≥36
All participants		243 (100)	16 (6.6)	35 (14.4)	69 (28.4)	123 (50.6)
Gender	Male	146 (60.1)	11 (7.5)	25 (17.1)	36 (24.7)	74 (50.7)
	Female	97 (39.9)	5 (5.2)	10 (10.3)	33 (34.0)	49 (50.5)
Disability conditions	MD	108 (44.4)	6 (5.6)	15 (13.9)	38 (35.2)	49 (45.4)
	MD + CP	55 (22.6)	8 (14.5)	8 (14.5)	13 (23.6)	26 (47.3)
	MD + A	14 (5.8)	1 (7.1)	4 (28.6)	1 (7.1)	8 (57.1)
	DS	36 (14.8)	0 (0.0)	2 (5.6)	9 (25.0)	25 (69.4)
	MD + S & HI	12 (4.9)	0 (0.0)	2 (16.7)	4 (33.3)	6 (50.0)
	MD + LD	18 (7.4)	1 (5.6)	4 (22.2)	4 (22.2)	9 (50.0)
Intelligence quotient	Borderline MD	5 (2.1)	0 (0.0)	1 (2.9)	3 (4.3)	1 (0.8)
	Mild MD	90 (37.0)	9 56.2)	15 (42.9)	30 (43.5)	36 (29.3)
	Moderate MD	100 (41.2)	3 (18.8)	12 (34.3)	25 (36.2)	60 (48.8)
	Severe MD	41 (16.9)	4 (25.0)	7 (20.0)	10 (14.5)	20 (16.3)
	Profound MD	7 (2.9)	0 (0.0)	0 (0.0)	1 (1.4)	6 (4.9)

MD Mental disability, CP Cerebral palsy, DS Down’s syndrome, LD Learning disability, A Autism, S-HI Sensory-hearing impairment.

The distribution of DAI scores, according to each of its components that are listed by the disability condition is given in Table 2. Incisal segment crowding (84.8%) was the most common aspect of malocclusion, followed by a largest anterior mandibular irregularity of ≥ 1 mm (77.8%) and largest anterior maxillary irregularity of ≥ 1 mm (68.3%). Anterior mandibular overjet (n = 15; 6.2%) was the least common aspect of malocclusion among the children surveyed. The entire group of children with DS had 1 or 2 incisal segment crowding, 97.2% (n = 35) had a largest anterior maxillary irregularity of ≥ 1 mm and 88.9% (n = 32) had a full cusp deviation in the antero-posterior molar relationship. Approximately 81% (n = 87) of the children with MD had a largest anterior mandibular irregularity of ≥ 1 mm and 75.9% (n = 82) had an anterior maxillary overjet ≥3 mm.

**Table 2:** **Distribution of DAI components according to various disability conditions**

DAI components		MD	MD + CP	MD + A	DS	MD + S-HI	MD + LD	Total	P value
Missing teeth	0	94 (87.0)	50 (90.9)	13 (92.9)	26 (72.2)	9 (75)	18 (100)	210 (86.4)	0.037
	≥1	14 (13.0)	5 (9.1)	1 (7.1)	10 (27.8)	3 (25)	0 (0)	33 (13.6)	
Incisal segment crowding	0	15 (13.9)	12 (21.8)	7 (50)	0 (0)	1 (8.3)	2 (11.1)	37 (15.2)	<0.001
	1-2	93 (86.1)	43 (78.2)	7 (50)	36 (100)	11 (91.7)	16 (88.9)	206 (84.8)	
Incisal segment spacing	0	46 (42.6)	27 (49.1)	7 (50)	18 (50)	6 (50)	9 (50)	113 (46.5)	0.944
	1-2	62 (57.4)	28 (50.9)	7 (50)	18 (50)	6 (50)	9 (50)	130 (53.5)	
Midline diastema	0	72 (66.7)	45 (81.8)	8 (57.1)	31 (86.1)	8 (66.7)	14 (77.8)	178 (73.3)	0.081
	≥1 mm	36 (33.3)	26 (47.3)	7 (50)	35 (97.2)	10 (83.3)	13 (72.2)	166 (68.3)	
Largest anterior maxillary irregularity	0	36 (33.3)	26 (47.3)	7 (50)	1 (2.8)	2 (16.7)	5 (27.8)	77 (31.7)	<0.001
	≥1 mm	72 (66.7)	29 (52.7)	7 (50)	35 (97.2)	10 (83.3)	13 (72.2)	166 (68.3)	
Largest anterior mandibular irregularity	0	21 (19.4)	17 (30.9)	6 (42.9)	6 (16.7)	1 (8.3)	3 (16.7)	54 (22.2)	0.124
	≥1 mm	87 (80.6)	38 (69.1)	8 (57.1)	30 (83.3)	11 (91.7)	15 (83.3)	189 (77.8)	
Anterior maxillary overjet	0-2 mm	26 (24.1)	16 (29.1)	2 (14.3)	24 (66.7)	4 (33.3)	6 (33.3)	78 (33.1)	<0.001
	≥3 mm	82 (75.9)	39 (70.9)	12 (85.7)	12 (33.3)	8 (66.7)	12 (66.7)	165 (67.9)	
Anterior mandibular overjet	0	104 (96.3)	52 (94.5)	13 (92.9)	30 (83.3)	11 (91.7)	18 (100)	228 (93.8)	0.096
	≥1 mm	4 (3.7)	3 (5.5)	1 (7.1)	6 (16.7)	1 (8.3)	0 (0)	15 (6.2)	
Vertical anterior openbite	0	91 (84.3)	44 (80)	12 (85.7)	25 (69.4)	11 (91.7)	15 (83.3)	198 (81.5)	0.401
	≥1 mm	17 (15.7)	11 (20)	2 (14.3)	11 (30.6)	1 (8.3)	3 (16.7)	45 (18.5)	
Antero-posterior molar relationship	Normal	20 (18.5)	9 (16.4)	2 (14.3)	2 (5.6)	2 (16.7)	1 (5.6)	36 (14.8)	<0.001
	Half cusp deviation	50 (46.3)	25 (45.5)	5 (35.7)	2 (5.6)	2 (16.7)	6 (33.3)	90 (37)	
	Full cusp deviation	38 (35.2)	21 (38.2)	7 (50)	32 (88.9)	8 (66.7)	11 (61.1)	117 (48.1)	

MD Mental disability, CP Cerebral palsy, DS Down’s syndrome, LD Learning disability, A – Autism, S-HI Sensory-hearing impairment.

The mean DMFT score was 4.36 ± 3.81, and 82.8% of the adolescents had a DMFT score >0 (Table 3). Significant differences in the DMFT scores were found between the various disability conditions (p < 0.05). Children with a co-existing MD and autism had a significantly lower mean DMFT score compared to children with other disability conditions. A highly significant difference in the DMFT scores was found between the institutionalized children and those residing with their parents (p < 0.001). Institutionalized children had a significantly lower mean DMFT score compared to children residing with their parents. No significant correlation was observed between the mean DAI scores and the mean DMFT scores (r = 0.090, p = 0.15).

**Table 3:** **DMFT scores according to the variables (gender, residential status, disability conditions, intelligence quotient and DAI scores)**

Variables		DMFT (Mean ± SD)	p value
Gender	Male	4.04 ± 3.74	> 0.05
	Female	4.85 ± 3.90	
Residential status	Resident	3.78 ± 3.09	<0.001
	Non-resident	4.76 ± 4.21	
Disability conditions	MD	4.05 ± 3.53	0.03
	MD + CP	4.11 ± 3.83	
	MD + A	2.07 ± 2.43	
	DS	5.61 ± 4.56	
	MD + S-HI	5.63 ± 3.32	
	MR + LD	4.75 ± 4.15	
Intelligence quotient	Borderline + mild MD	4.24 ± 3.88	0.49
	Moderate MD	4.66 ± 3.74	
	Severe + profound MD	3.92 ± 3.86	
DAI scores	≤ 25	3.59 ± 2.62	0.15
	26 – 30	3.72 ± 4.57	
	31 – 35	4.68 ± 3.67	
	≥ 36	4.45 ± 3.80	

MD Mental disability, CP Cerebral palsy, DS Down’s syndrome, LD Learning disability, A Autism, S-HI Sensory-hearing impairment.

## Discussion

This study describes the prevalence of malocclusion among 12-18-year-old CSHCN in Chennai, India, and the association between malocclusion and dental caries among these children. The DAI has been adopted by the World Health Organization (WHO) in an attempt to establish a simple and universally acceptable orthodontic index for use in epidemiological surveys [20] and has been reported to be reliable and valid for determination of orthodontic treatment needs [21]. Because it is easy to use and identifies abnormal occlusal traits [22] and because it was validated in India [23], the DAI was used in our study.

The reported prevalence of malocclusion in India ranges from 20% to 43% [24], and among disabled individuals the prevalence is 47% [11]. However, the results of the present study indicate that the prevalence of malocclusion among the surveyed disabled children in the age group of 12–18 years was approximately 93%. The percentage of participants with severe malocclusion (28.4%) and handicapping malocclusion (50.6%) was much higher than that reported in a previous Indian study (12.2% and 10.6%, respectively) [11]. The results of our study are not in accordance with a Nigerian study [12] that reported a 47% prevalence of malocclusion among disabled children in the age group of 12–18 years. Furthermore, the prevalence of malocclusion among disabled children was much higher than those reported in a recent south Indian study among 1800 normal school children aged 11 – 15 years using DAI [14].

Vittek et al. [25] reported that individuals with MD had a higher proportion of Class III malocclusion compared to controls. Vigild [26] reported 23% anterior open bites and 29% anterior crossbite among mentally subnormal adolescents. Among the sample of children with MD alone, 35.2% had full cusp deviation in the antero-posterior molar relationship. A study conducted by Onyeaso [27] reported that 32% of individuals with MD had a handicapping malocclusion, whereas in our study, 45.4% of individuals with MD had a handicapping malocclusion. Dinesh et al. [11] reported that the prevalence of a handicapping malocclusion is increased, to some extent, with an increased severity of MD (13.5% to 15.3%). Furthermore, some studies have reported that the prevalence of an anterior open bite increased slightly with a corresponding increase in the severity of MD [11, 25, 28]. A similar trend in the prevalence of an anterior open bite was observed in the present study; 15.6% children with mild MD, 19% with moderate MD and 22% with severe MD had a vertical anterior open bite ≥ 1 mm. A review on malocclusion among individuals with mental and physical disabilities concluded that malocclusion was higher in individuals with disabilities [2]. According to Muppa et al. [29] the prevalence of malocclusion in individuals with special needs is associated with the type of disability and it is more in males. Mentally disabled individuals had higher frequencies of all types of malocclusion. Another study on the oral health status of children with special health care needs showed that majority of the children had poor oral hygiene with high caries prevalence and moderate gingivitis [30, 31].

Previous studies have reported that missing teeth, anterior diastema and Class II malocclusions were most common among individuals with CP [13, 32]. In our sample of children with MD and CP, 9.1% had ≥1 missing teeth; 47.3% had midline diastema; and 45.5% and 38.2% had a half- and full-cusp deviation, respectively, in the antero-posterior molar relationship. The common malocclusion traits among individuals with CP have been attributed to the early eruption of primary teeth and an aberrant tongue and head posture [28, 32]. Furthermore, an excessive maxillary overjet in individuals with CP have been reported to be the caused by lip incompetence and failure of the maxillary orbicularis muscle [10, 13, 32].

According to a study conducted by Shyama et al. [13] 48.6% of individuals with DS had a slight malocclusion, whereas 36.6% had severe malocclusion. However, among our sample of children with DS, 25% had a severe malocclusion, whereas 69.4% had a handicapping malocclusion. The total number of individuals with DS included in the study conducted by Shyama et al. [13] was 183, whereas the total number of children with DS in the present study was 36 and hence the comparison should be made with caution. Several studies have reported a high prevalence of negative overjet (36.4% to 41%), anterior open bite (4.9% to 63.6%) and posterior crossbite (14.6% to 65%) among individuals with DS compared to both other disabled individuals and to controls [25, 26, 28]. Among our sample of children with DS, 16.7% had an anterior mandibular overjet ≥ 1 mm and 30.6% had a vertical anterior open bite ≥ 1 mm. In addition, all individuals with DS had incisal segment crowding, and 88.9% had full cusp deviation in the antero-posterior molar relationship. DS was reported as a significant risk factor for severe malocclusion and has been attributed to altered cranial-base relationships [13], diminished dental arch size, decreased arch length and the reduced maxillary size characteristic of DS [13, 28, 33].

Crowding, anterior diastema and more than a half-cusp deviation of the antero-posterior molar relationship were reported as the common malocclusion traits among disabled individuals by Dinesh et al. [11] and Onyeaso [12]. Incisal segment crowding and largest anterior mandibular and maxillary irregularities ≥1 mm were all common among our surveyed sample of disabled children.

Stabholz [34] reported that the residential status of individuals with MD may have variable effects on their oral health status. The mean DMFT score of the non-resident children was significantly higher compared to the score of the residents of institutions in this study. A significant difference in the mean DMFT scores between children with various disability conditions was also noticed. A study conducted by Al-Qahtani and Wayne [35] reported a mean DMFT score of 3.80 ± 1.67, 5.12 ± 3.45 and 5.81 ± 2.95 among 11-12-year-old blind, deaf and mentally disabled children, respectively. Purohit et al. [36] reported a mean DMFT score of 2.52 ± 2.61 for CSHCN and a mean DMFT score of 0.61 ± 1.12 for healthy controls. The authors also reported that 66.4% of CSHCN had definite malocclusion (DAI score 26 – 30), 10.9% had severe malocclusion (DAI score 31 – 35) and 0.4% had handicapping malocclusion (DAI score ≥ 36), whereas 82.6% of healthy controls had no abnormality or only minor malocclusion (DAI score ≤ 25) and 17.4% had definite malocclusion. The mean DMFT score was 4.36 ± 3.81, and 50.6% of the participants had DAI scores ≥ 36. Previous studies have reported a significant association between DAI scores of ≥36 and high caries among healthy children [14, 37]. However, no significant association was found between DAI scores and the prevalence of caries among our sample of disabled children. Stahl and Grabowski [38] also concluded that no significant association between malocclusion and dental caries in the mixed dentition period could be drawn from their study.

Some limitations of the present study should be considered when interpreting the results. Ondarza et al. [33] reported that there are questions about the reliability of the clinical measures of malocclusion, such as the DAI, the Index of Orthodontic Treatment Need (IOTN) and Angle’s classification of malocclusion. The esthetic and anatomic components of malocclusion are included in both the DAI and the IOTN [39], but the IOTN is reported to be more precise [37]. DAI was preferred in our study because previous studies conducted among healthy children [14, 15] and among disabled children [11, 36] have used this index to report the prevalence of malocclusion in India. Visible proximal caries, loss of inter-proximal translucency and ‘catch’ during inter-proximal probing were included as the clinical criteria to diagnose proximal caries, but bitewing radiographs were not taken to determine proximal caries in our study. According to the WHO Oral Health Survey Basic Methods 1997 [18], radiographs are not required and dental caries are measured using dentition status and treatment needs, which covers both primary dentition and permanent dentition along with root status. Ethical, feasibility and logistics issues were also considered in the decision not to take bitewing radiographs in this study. The data regarding the general status such as disability status, IQ, systemic diseases and history of regular medications collected from the medical records of the participants might have slightly influenced the outcome of the study [40].However due to the time and logistic constrains we were not able to evaluate the condition along with the oral examination. Our sample of children with various disabilities was not evenly distributed and thus some of the disabilities are not well represented. In addition, healthy age-matched control subjects were not included, therefore a comparative analysis of the prevalence of malocclusion was not possible.

## Conclusion

Within the limitations of this study, the prevalence of malocclusion and dental caries appeared to be high among the disabled children surveyed. An incisal crowding and anterior maxillary and mandibular irregularity of ≥ 1 mm were the most common orthodontic anomalies in these children. However, there was no positive correlation between the severity of malocclusion and the presence of dental caries.

## References

[1] de Nova-Garcia MJ, Martinez MR, Sanjuan CM, Lopez NE, Cabaleiro EC, Garcia YA (2007). Program for coordinated dental care under general anaesthesia for children with special needs. Med Oral Patol Oral Cir Bucal.

[2] Winter K, Baccaglini L, Tomar S (2008). A review of malocclusion among individuals with mental and physical disabilities. Spec Care Dentist.

[3] AAPD (2012). Definition of special health care needs -council on clinical affairs. Reference Manual.

[4] Hennequin M, Moysan V, Jourdan D, Dorin M, Nicolas E (2008). Inequalities in oral health for children with disabilities: a French national survey in special schools. PLoS One.

[5] Macnab AJ, Rozmus J, Benton D, Gagnon FA (2008). 3-year results of a collaborative school-based oral health program in a remote First Nations community. Rural Remote Health.

[6] Santos MT R d, Masiero D, Novo NF, Simionato MR (2003). Oral conditions in children with cerebral palsy. J Dent Child (Chic).

[7] Weng RH, Kung PT, Tsai WC, Chiang HH, Chiu LT (2011). The use of fluoride varnish and its determining factors among children with disability in Taiwan. Res Dev Disabil.

[8] Gardens SJ, Krishna M, Vellappally S, Alzoman H, Halawany HS, Abraham NB, Jacob V (2013). Oral health survey of 6-12-year-old children with disabilities attending special schools in Chennai, India. Int J Paediatr Dent.

[9] Northway WM, Wainright RL, Demirjian A (1984). Effects of premature loss of deciduous molars. Angle Orthod.

[10] Franklin DL, Luther F, Curzon ME (1996). The prevalence of malocclusion in children with cerebral palsy. Eur J Orthod.

[11] Dinesh RB, Arnitha HM, Munshi AK (2003). Malocclusion and orthodontic treatment need of handicapped individuals in South Canara. India Int Dent J.

[12] Onyeaso CO (2004). Comparison of malocclusions and orthodontic treatment needs of handicapped and normal children in Ibadan using the Dental Aesthetic Index (DAI). Niger Postgrad Med J.

[13] Shyama M, Al-Mutawa SA, Honkala S (2001). Malocclusions and traumatic injuries in disabled schoolchildren and adolescents in Kuwait. Spec Care Dentist.

[14] Baskaradoss JK, Geevarghese A, Roger C, Thaliath A (2013). Prevalence of malocclusion and its relationship with caries among school children aged 11–15 years in southern India. Korean J Orthod.

[15] Singh A, Purohit B, Sequeira P, Acharya S, Bhat M (2011). Malocclusion and orthodontic treatment need measured by the dental aesthetic index and its association with dental caries in Indian schoolchildren. Community Dent Health.

[16] McPherson M, Arango P, Fox H, Lauver C, McManus M, Newacheck PW, Perrin JM, Shonkoff JP, Strickland B (1998). A new definition of children with special health care needs. Pediatrics.

[17] **Census data 2001** [http://www.censusindia.gov.in/2011-common/census_data_2001.html]

[18] WHO (1997). Oral health surveys: basic methods.

[19] Dunning JM (1986). Principles of dental public health.

[20] Jenny J, Cons NC, Kohout FJ, Jakobsen J (1993). Predicting handicapping malocclusion using the Dental Aesthetic Index (DAI). Int Dent J.

[21] Jenny J, Cons NC (1996). Comparing and contrasting two orthodontic indices, the Index of Orthodontic Treatment need and the Dental Aesthetic Index. Am J Orthod Dentofacial Orthop.

[22] Onyeaso CO, Aderinokun GA (2003). The relationship between dental aesthetic index (DAI) and perceptions of aesthetics, function and speech amongst secondary school children in Ibadan, Nigeria. Int J Paediatric Dentistry/British Paedodontic Soc Int Assoc Dentistry Children.

[23] Poonacha KS, Deshpande SD, Shigli AL (2010). Dental aesthetic index: applicability in Indian population: a retrospective study. J Indian Soc Pedod Prev Dent.

[24] Sureshbabu A, Chandu G, Shafiulla M (2005). Prevalence of malocclusion and orthodontic treatment needs among 13–15 year old school going children of Davangere city, Karnataka, India. J Indian Assoc Public Health Dent.

[25] Vittek J, Winik S, Winik A, Sioris C, Tarangelo AM, Chou M (1994). Analysis of orthodontic anomalies in mentally retarded developmentally disabled (MRDD) persons. Spec Care Dentist.

[26] Vigild M (1985). Prevalence of malocclusion in mentally retarded young adults. Community Dent Oral Epidemiol.

[27] Onyeaso CO (2003). Orthodontic treatment need of mentally handicapped children in Ibadan, Nigeria, according to the dental aesthetic index. J Dent Child (Chic).

[28] Oreland A, Heijbel J, Jagell S (1987). Malocclusions in physically and/or mentally handicapped children. Swed Dent J.

[29] Muppa R, Bhupathiraju P, Duddu MK, Dandempally A, Karre DL (2013). Prevalence and determinant factors of malocclusion in population with special needs in South India. J Indian Soc Pedod Prev Dent.

[30] Saravanakumar M, Vasanthakumari A, Bharathan R (2013). Oral health status of special health care needs children attending a day care centre in Chennai. Int Students’ Res.

[31] Namal N, Vehit HE, Koksal S (2007). Do autistic children have higher levels of caries? A cross-sectional study in Turkish children. J Indian Soc Pedod Prev Dent.

[32] Strodel BJ (1987). The effects of spastic cerebral palsy on occlusion. ASDC J Dent Child.

[33] Ondarza A, Jara L, Bertonati MI, Blanco R (1995). Tooth malalignments in Chilean children with Down syndrome. The Cleft palate-craniofacial J: off Pub Am Cleft Palate-Craniofacial Assoc.

[34] Stabholz A, Mann J, Sela M, Schurr D, Steinberg D, Shapira J (1991). Caries experience, periodontal treatment needs, salivary pH, and Streptococcus mutans counts in a preadolescent Down syndrome population. Spec Care Dentist.

[35] Al-Qahtani Z, Wyne AH (2004). Caries experience and oral hygiene status of blind, deaf and mentally retarded female children in Riyadh, Saudi Arabia. Odonto-stomatologie tropicale = Tropical dental J.

[36] Purohit BM, Acharya S, Bhat M (2010). Oral health status and treatment needs of children attending special schools in South India: a comparative study. Spec Care Dentist.

[37] Gabris K, Marton S, Madlena M (2006). Prevalence of malocclusions in Hungarian adolescents. Eur J Orthod.

[38] Stahl F, Grabowski R (2004). Malocclusion and caries prevalence: is there a connection in the primary and mixed dentitions?. Clin Oral Investig.

[39] Manzanera D, Montiel-Company JM, Almerich-Silla JM, Gandia JL (2010). Diagnostic agreement in the assessment of orthodontic treatment need using the Dental Aesthetic Index and the Index of Orthodontic Treatment Need. Eur J Orthod.

[40] Nagurney JT, Brown DF, Sane S, Weiner JB, Wang AC, Chang Y (2005). The accuracy and completeness of data collected by prospective and retrospective methods. Acad Emergency Med: Off J Soc Acad Emergency Med.

[CR41] The pre-publication history for this paper can be accessed here:http://www.biomedcentral.com/1472-6831/14/123/prepub

